# Use of Pulsed-Field Gel Electrophoresis to Determine the Source of Methicillin-Resistant *Staphylococcus aureus* Bacteremia

**DOI:** 10.3390/idr13030056

**Published:** 2021-06-25

**Authors:** Eugene Y. H. Yeung, Ivan Gorn

**Affiliations:** 1 Department of Medical Microbiology, The Ottawa Hospital General Campus, The University of Ottawa, Ottawa, ON K1H 8L6, Canada; 2 The Ottawa Hospital Research Institute, Ottawa, ON K1H 8L6, Canada; igorn@ohri.ca

**Keywords:** pulsed-field gel electrophoresis, methicillin-resistant *Staphylococcus aureus*, bacteremia, colonoscopy, pelvic trauma, source control of infection, screening, clonality, molecular microbiology

## Abstract

Pulsed-field gel electrophoresis (PFGE) has historically been considered the gold standard in fingerprinting bacterial strains in epidemiological studies and outbreak investigations; little is known regarding its use in individual clinical cases. The current study detailed two clinical cases in which PFGE helped to determine the source of their methicillin-resistant *Staphylococcus aureus* (MRSA) bacteremia. Patient A was found to have MRSA bacteremia after trauma in her pelvic area. MRSA was also found in her groin but not in her nostril and rectum. PFGE was performed that showed variable bands of her MRSA isolates from blood and groin, suggestive of different strains of MRSA. Her MRSA bacteremia was determined to be unrelated to her pelvic trauma. Patient B was found to have MRSA bacteremia after colonoscopy. MRSA was also found in his nostril and rectum. PFGE was performed that showed variable bands of his MRSA isolates from blood and rectum but identical bands of MRSA isolates from his blood and nostril. His MRSA bacteremia was determined to be unrelated to his colonoscopy procedure. The current study demonstrates the use of PFGE to rule out the source of bacteremia in individual clinical cases.

## 1. Introduction

Pulsed-field gel electrophoresis (PFGE) has historically been considered the gold standard in fingerprinting bacterial strains in epidemiological studies and outbreak investigations in healthcare settings [1]. PFGE has been shown to have superior discriminatory powers compared to antibiograms [2]. Although PFGE has been widely used in epidemiological studies, little is known regarding its use in individual clinical cases. A PFGE study was performed on 29 patients with recurrent *Staphylococcus aureus* bacteremia, defined as a subsequent episode of *S. aureus* bacteremia after completion of an antimicrobial course of therapy yielding an apparent clinical cure [3]. Patients with PFGE-confirmed relapse were more likely to have an indwelling foreign body, with an odds ratio 18.2 (95% confidence interval 7.6–43.6, *p* < 0.001). The results suggested that PFGE findings may impact on patients’ clinical management, such as removal of foreign bodies for source control.

Identifying the source in a patient’s methicillin-resistant *Staphylococcus aureus* bacteremia (MRSA) helps to determine the choice and duration of antimicrobials. For example, in bacterial native vertebral osteomyelitis, a total duration of 6 weeks of parenteral or highly bioavailable oral antimicrobial therapy is recommended [4]. In native valve endocarditis caused by MRSA, a total duration of 6 weeks of vancomycin is recommended; in contrast, in prosthetic valve endocarditis caused by MRSA, a minimum of 6 weeks of vancomycin and rifampin, plus 2 weeks of gentamicin, is recommended [5]. Even if a patient’s *S. aureus* bacteremia appears to be due to contamination, it should be treated as a true bloodstream infection with at least 2 weeks of antimicrobials [6]. If PFGE can identify the source of patients’ bacteremia, clinicians may be more confident to discontinue antimicrobials early to preserve patients’ microbiome.

In the Ottawa Hospital, our infectious disease team encountered two patients whose source of MRSA bacteremia was initially unclear. The MRSA screening swabs on their groin and rectum were positive, respectively, suggesting these to be the source of infection. Using PFGE, our microbiology team helped to rule out the hypotheses on the source of the infections.

## 2. Materials and Methods

### 2.1. Data Set Creation

The current quality improvement project followed the guidelines and standards given by the Ottawa Health Science Network Research Ethics Board. The data in the current study were collected from established laboratory methodologies. The current case series was based on two patients with MRSA bacteremia encountered by the Ottawa Hospital Infectious Diseases team. The patients kindly provided written consents for their clinical and laboratory information to be included in the current manuscript. Each patient’s electronic health record (EHR) was retrospectively reviewed using the software Epic Hyperspace (Version November 2018, Verona, MI, USA). Their demographics, clinical history, and therapeutic records were recorded on a separate spreadsheet. Their laboratory information was obtained from the Eastern Ontario Regional Laboratory Association (EORLA), which used the Cerner Millennium software (Version 2013.04.1.34; Kansas City, MO, USA) to store patients’ data.

### 2.2. Detection of Microorganims in Positive Blood Culture Bottles

On the hospital wards, the patients’ blood samples were collected, transferred to BD BACTEX aerobic and anaerobic fluid culture vials, and incubated in the microbiology laboratory using established clinical laboratory tools. When the BD BACTEX FX automated blood culture incubator detected growth in blood cultures, the blood culture bottles were removed from the incubator. Drops from the blood culture bottles were used for direct Gram stain. When Gram-positive cocci were detected on Gram stain, drops from blood culture bottles were incubated in blood and chocolate agar plates in a carbon dioxide incubator for 48 h, MacConkey agar plates in an oxygen incubator for 48 h, and CDC anaerobic blood agar plates in an anaerobic incubator for 48 h, all at 35 °C. The microorganisms isolated from the above agar plates were further identified using Bruker matrix-assisted laser desorption ionization-time of flight mass spectrometry (MALDI-TOF MS). Each microorganism was identified to species level when the MALDI-TOF score was ≥2.0.

### 2.3. Identification of MRSA Isolates in Blood Cultures

When *Staphylococcus aureus* isolates were identified with MALDI-TOF MS, antimicrobial susceptibility testing was performed in the BD Phoenix automated identification and susceptibility testing system. Antimicrobial susceptibility testing with cefoxitin, oxacillin, rifampin, sulfamethoxazole-trimethoprim, and vancomycin was performed on MRSA isolates from blood cultures and interpreted with the Clinical and Laboratory Standards Institute (CLSI) minimum inhibitory concentration breakpoints. When the cefoxitin minimum inhibitory concentration was ≥8 µg/mL, a rapid latex agglutination assay (Staphytect Plus, Oxoid Plus, Basingstoke, UK) would be performed that detects penicillin-binding protein 2a in the isolates. A positive reaction confirms MRSA strain.

### 2.4. Identification of MRSA in Screening Swabs

MRSA screening was performed by swabbing a patient’s nostril, groin, and rectum using Copan ESwab Collection and Transport System. The swabs were transferred to the microbiology laboratory and cultured on chromogenic agar (Denim Blue) plates at 35 °C in an oxygen incubator for 24 h. Growth of blue colonies on the denim blue plates were worked up for MRSA identification by rapid latex agglutination assay. Antimicrobial susceptibility testing was not routinely performed on MRSA isolates identified from screening swabs.

### 2.5. PFGE

A PFGE analysis was performed on the MRSA isolates using the CHEF Mapper (Bio-Rad, Hercules, CA, USA) with previously validated protocols [7,8]. Agarose gel and cell lysis, plug lysis, protein kinase, and buffer solutions were prepared as per the established protocols [7]. For discrimination of *Staphylococcus* species, Sma I restriction enzyme was used to digest the DNA material at room temperature for 4 h. The CHEF Mapper was preset to have a voltage of 6 V/cm and initial switch time, final switch time, and run time of 5 s, 35 s, and 20 h, respectively. The strain spectra were visually examined to identify peaks with variable occurrence among strains, followed by hierarchical clustering with the online software DendroUPGMA (http://genomes.urv.cat/UPGMA/)(accessed on 25 April 2021). The Unweighted Pair Group Method with Arithmetic Mean (UPGMA) with Pearson correlation coefficient was used to compare between sets of variables in dendrogram. Strain relatedness was determined using the Tenover criteria [9], in which 0 fragment difference suggested indistinguishable strains, 2–3 fragment differences suggested closely related strains, 4–6 fragment differences suggested possibly related strains, and ≥7 fragment differences suggested different strains.

## 3. Results

Patient A was a 40-year-old female with no significant past medical history admitted to hospital after being hit by an all-terrain vehicle and intentionally stabbed in the right groin and left lateral thigh. She suffered fracture of superior and inferior pubic rami bilaterally with communition and connection into the left acetabulum. Her injury was treated conservatively with no operative procedures. On day 8 after the trauma, patient was found to have tachycardia but no fever. A doppler ultrasound of her bilateral upper and lower extremities and computed tomography pulmonary angiogram imaging were performed that showed no signs of thromboembolism. On day 12, the diagnosis of her tachycardia was not established, and therefore two sets of blood cultures were collected. The first set of blood cultures showed Gram-positive cocci in clusters after about 15 h of incubation in each of the aerobic bottle and anaerobic bottle. The other blood culture set showed Gram-positive cocci in clusters bottle after about 15.5 and 21.5 h of incubation in the anaerobic and aerobic bottles, respectively. Two sets of repeat blood culture were drawn on day 13, prior to intravenous vancomycin being initiated. The microorganism in blood culture was later identified to be MRSA, resistant to cefoxitin and oxacillin, and susceptible to rifampin, sulfamethoxazole-trimethoprim, and vancomycin. No other microorganisms were identified in the four blood culture bottles on day 12 after 120 h of incubation. The repeat blood culture on day 13 consistently showed MRSA after 14.5 and 20 h of incubation in two anaerobic bottles, respectively. Two more sets of blood culture were collected 3 days after the initial blood culture (day 15 after trauma) and eventually showed no growth after 120 h. MRSA screening swab from her groin, but not from her nostril and rectum, also showed MRSA. Her transthoracic and transesophageal echocardiogram showed no signs of endocarditis or vegetation. It was suspected that the source of her MRSA bacteremia was from translocation of microorganism through the stabbing trauma in her groin. The infectious diseases team requested PFGE analysis on her groin and blood MRSA isolates to determine the strain relatedness. Unfortunately, the PFGE analysis had to be postponed due to redeployment of laboratory workforce to help with COVID-19 testing. Patient A received 4 more weeks of intravenous vancomycin (with target trough 15–20 mg/L) after the first negative blood culture on day 15. She was followed up in the infectious diseases clinic at the end of her vancomycin therapy. Patient’s repeat blood culture after discontinuation of vancomycin showed no growth. She was clinically stable and was therefore discharged from the clinic.

Patient B was a 72-year-old male, with history of long-term immunosuppressant (sirolimus and prednisone) use for heart transplant, hormonal therapy use for hypogonadism, bronchiectasis, toxic thyroid nodules, and chronic kidney disease, admitted to hospital because of repeat episodes of bright red blood per rectum. Stool culture, *Clostridioides difficile*, and ova and parasite testing did not identify an infectious etiology. A screening swab collected from his nostril showed he was an MRSA colonizer, but he did not complain of any nasal symptoms. On day 2 of his hospital admission, a colonoscopy was performed that showed severe diverticulosis of his colon. On day 4, Patient B had a body temperature of 39 °C that prompted two sets of blood cultures being collected, followed by administration of intravenous vancomycin and piperacillin-tazobactam. One set of blood cultures showed Gram-positive cocci in clusters in the aerobic bottle after 18 h of incubation. The other set of blood cultures showed Gram-positive cocci in clusters in the aerobic and anaerobic bottles after about 24.5 and 25 h of incubation, respectively. The microorganism in blood cultures was later identified to be MRSA, resistant to cefoxitin and oxacillin, and susceptible to rifampin, sulfamethoxazole-trimethoprim, and vancomycin. His vancomycin was continued and piperacillin-tazobactam was discontinued. No other microorganisms were identified in the four blood culture bottles after 120 h of incubation. Two sets of repeat blood culture were collected 2 days after the initial blood culture (day 6 of admission) and eventually showed no growth after 120 h. MRSA screening swabs from his nostril and rectum also showed MRSA. His transthoracic and transesophageal echocardiogram showed no signs of endocarditis or vegetation. It was suspected that the source of his MRSA bacteremia was through translocation of microorganism through his recent colonoscopy. Another differential was from his recent thrombophlebitis in left cephalic vein where an intravenous canula was used for blood transfusion. The infectious diseases team requested PFGE analysis on his rectal and blood MRSA isolates to determine the strain relatedness. Unfortunately, the PFGE analysis had to be postponed due to redeployment of laboratory workforce to help with COVID-19 testing. Patient B received 4 more weeks of intravenous vancomycin (with target trough 15–20 mg/L) after the first negative blood culture on day 6. He was followed up in the infectious diseases clinic at the end of his vancomycin therapy. Patient had subjectively improved, and therefore no repeat blood culture was needed after discontinuation of vancomycin therapy. He was then discharged from the clinic.

**Figure 1:**
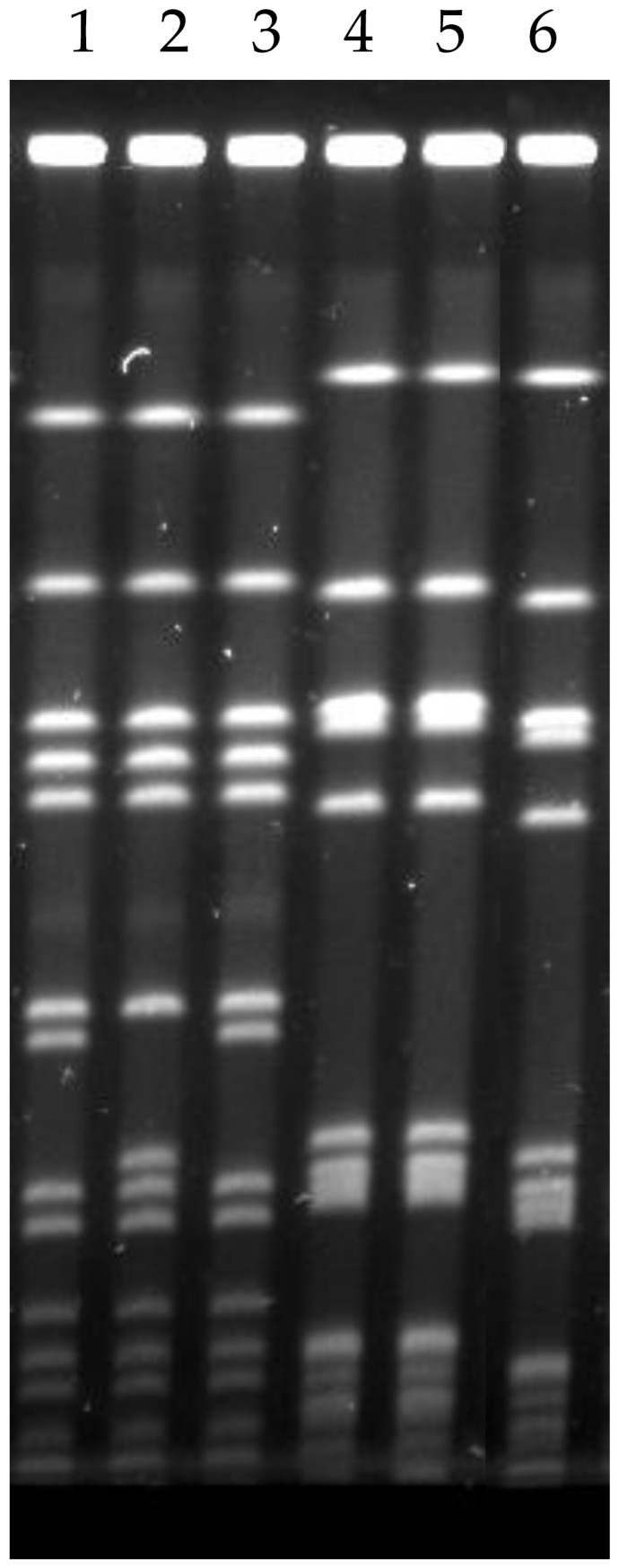
Pulsed-field gel electrophoresis (PFGE). Lanes 1–3 are Patient A’s (pelvic trauma patient) methicillin-resistant *Staphylococcus aureus* (MRSA) isolates from the first set of blood cultures (day 12), groin swab, and third set of blood cultures (day 13), respectively. Lanes 4–6 are Patient B’s (colonoscopy patient) MRSA isolates from nasal swab, blood culture, and rectal swab, respectively.

**Figure 2:**
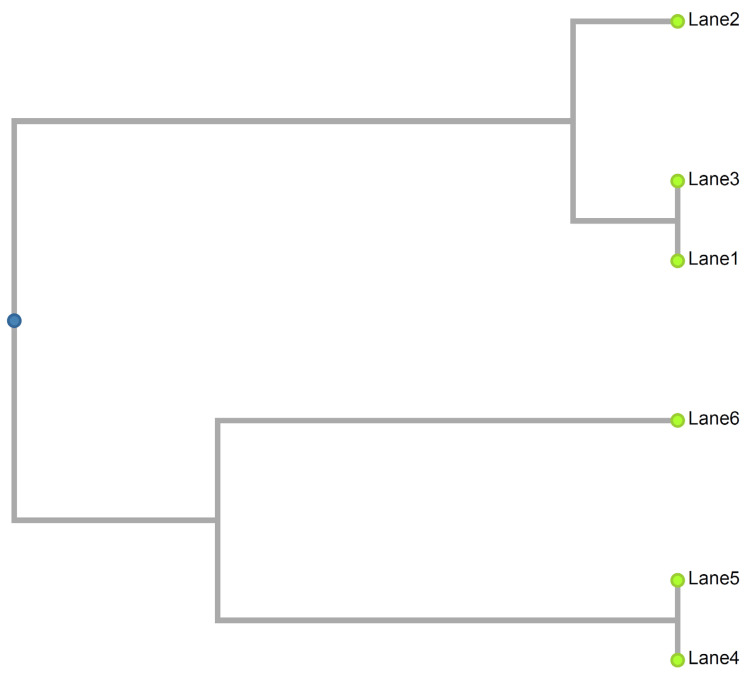
Dendrogram based on the pulsed-field gel electrophoresis (PFGE) results of all 6 methicillin-resistant *Staphylococcus aureus* (MRSA) isolates. Lanes 1–3 are Patient A’s (pelvic trauma patient) MRSA isolates from the first set of blood cultures (day 12), groin swab, and third set of blood cultures (day 13), respectively. Lanes 4–6 are Patient B’s (colonoscopy patient) MRSA isolates from nasal swab, blood culture, and rectal swab, respectively.

PFGE analysis was retrospectively conducted (Figure 1). Patient A’s blood MRSA isolates (Lanes 1 and 3) were indistinguishable from each other with no fragment difference but with two fragments different from her groin MRSA isolate (Lane 2). Patient B’s nasal and blood MRSA isolates (Lanes 4 and 5, respectively) were indistinguishable from each other, but were more than seven fragments different from his rectal MRSA isolate. Figure 2 shows a dendrogram based on the PFGE results of all six MRSA isolates.

## 4. Discussion

Patient A’s PFGE results suggested that her MRSA bacteremia did not originate from her groin injury. Although her groin MRSA isolate (Lane 2 in Figure 1 and Figure 2) were only two fragments different from her blood MRSA isolates (Lanes 1 and 3), we could not rule in whether the difference was due to mutation to two closely related strains during her hospital stay. Her source of MRSA bacteremia remained unknown. Despite that, it is important to note that pelvic injury on arrival in emergency department was identified as a risk factor for bacteremia (odds ratio 2.25, *p* = 0.038) in a study of 859 trauma patients [10].

Patient B’s PFGE results suggested that his MRSA bacteremia did not originate from translocation of bacteria through his recent colonoscopy. His blood and rectal MRSA isolates (Lanes 5 and 6 in Figure 1 and Figure 2) were more than seven fragments different, suggesting that they were different strains. His nasal and blood MRSA isolates (Lanes 4 and 5) were indistinguishable. Patient B’s MRSA bacteremia was possibly from a MRSA strain that colonized his normal flora, as his blood and nasal MRSA isolates appeared to be identical strain. It was possible that his bacteremia originated from his recent thrombophlebitis in left cephalic vein where an intravenous canula was used for blood transfusion. Unfortunately, a sample of his thrombophlebitis lesion could not be collected; we could not possibly rule in our other hypotheses on the source of bacteremia. Despite that, it is important to note that cases of *S. aureus* bacteremia after endoscopy had been reported in the past [11]. The current case patients had no implanted prostheses, evidence of endocarditis or metastatic foci, repeat positive blood culture ≥2 days after initial set, or clinical deterioration after initiation of appropriate antimicrobials. Their MRSA bacteremia episodes were deemed uncomplicated; therefore, 4 weeks of intravenous vancomycin therapy were sufficient [6].

PFGE has occasionally been used to help determine the source of infection in clinical cases. An 85-year-old female patient died of cardiogenic shock associated with *Staphylococcus lugdunensis* bacteremia and native mitral valve infective endocarditis [8]. Of note, she was found to have *S. lugdunensis* bacteremia secondary to a tunneled dialysis catheter 10 months prior. PFGE showed that the *S. lugdunensis* in her latest and previous episodes of bacteremia had indistinguishable fragments, indicative of originating from the same strain. It was proposed that her antimicrobial treatment 10 months ago failed to completely clear her *S. lugdunensis* infection.

It is important to note that even if the same strain of microorganism were identified from screening sites and blood cultures, it would not always prove where the source of infection was. It is possible that patients’ infections originate from microorganisms colonized in their flora [12,13]. In a surveillance study of 4131 *S. aureus* isolates collected from patients at 43 centers in the United States, PFGE identified USA300 and USA100 strains in 61% and 18% of isolates, respectively, suggesting the same strain of MRSA could appear in multiple patients with no epidemiological link [14]. As demonstrated in Patient B, he had no nasal symptoms and did not appear to contract MRSA bacteremia from nasal infection, despite having the same strains of MRSA in his nasal swab and blood culture. However, repeat PFGE analysis on the same specimens would be required to help us confidently rule out the potential source of the infection—in his case, rectal translocation.

Studies showed that MRSA colonizers were more prone to have subsequent MRSA infection. In a surveillance study of 545 patients, the risk ratio for MRSA bacteremia with MRSA colonization of their chronic ulcer was 16 (95% confidence interval 6–45) [15]. In a study of 903,348 patients admitted to intensive care unit, the odds ratio of MRSA infection within 365 days of acquiring MRSA colonization was 6.2 (95% confidence interval 5.7–6.8) [16]. Despite that, the current study failed to show the MRSA strain in screening swabs being identical to the MRSA strain in blood cultures of Patient A.

A major limitation of the current study was the small sample size. This quality improvement project was a hypothesis-generating pilot study at best. We hope the current study data would encourage researchers and clinicians to explore with a larger sample sizes, the clinical applicability of PFGE and relevance of MRSA isolates identified in screening swabs. The current study’s PFGE results were not promptly available prior to patients finishing their antimicrobial therapies. PFGE analysis is a time-consuming and labor-intensive method [17]. PFGE analysis typically requires 4 days to complete in our laboratory; each gel in the PFGE holds a maximum of 15 clinical isolates only. In the future, laboratorians should investigate methods for bacterial strain fingerprinting with quicker turnaround time and discriminatory power. Bacterial strain typing with MALDI-TOF is currently being developed that should give fast and high throughput results if validated [18,19]. Nevertheless, a previous study conducted in our laboratory failed to show sufficient discriminatory power with MALDI-TOF bacterial strain typing [20]. Despite its high concordance with epidemiological relatedness, PFGE does not discriminate isolates to the same degree achieved by whole genome sequencing (WGS) and is prone to inter-operator variability [17]. In a study that compared PFGE with WGS for fingerprinting vancomycin-resistant *Enterococcus faecium* (*n* = 19), MRSA (*n* = 17), and *Acinetobacter baumannii* (*n* = 15), 28.9% of isolates were indistinguishable by PFGE but were deemed nonclonal by WGS [21]. WGS has been proposed to be the new gold standard method in bacterial strain fingerprinting, despite some hurdles, such as more data needed for defining clonal lineages for a larger library of microorganisms [1]. We acknowledge the lack of quality control band and universal standard in our gel. However, each patient’s own MRSA isolates from multiple sites could serve as a positive control; the other patient’s MRSA isolates could serve as a negative control. In the future, laboratorians may consider repeating the PFGE analysis on the same specimens on a different week to ensure the precision of the results. Finally, it is important to note that mobile genetic elements, such as plasmids, can be readily transferred between strains and even across species of bacteria, leading to different fragment patterns in the PFGE results [1]. The different fragment patterns of two independent isolates do not always exclude a common source.

## 5. Conclusions

The current study demonstrated that PFGE has the potential to help determine the source of bacteremia in individual patients. However, PFGE may be helpful to rule out rather than rule in the source of MRSA infection. Although the data are currently inconclusive on the source of infection, this study illustrated the difficulty with PFGE and might prompt further studies on a quicker, higher throughput, and more discriminatory methodology. More data are needed to justify the clinical applicability of molecular microbiology and the relevance of MRSA screening swabs.

## Data Availability

The raw data presented in this study are available on request from the corresponding author. The data are not publicly available due to patient confidentiality.
